# ASPIRE Model for Treating Cannabis and Other Substance Use Disorders: A Novel Personalized-Medicine Framework

**DOI:** 10.3389/fpsyt.2014.00180

**Published:** 2014-12-08

**Authors:** Udi E. Ghitza

**Affiliations:** ^1^Center for the Clinical Trials Network, National Institute on Drug Abuse, National Institutes of Health, Bethesda, MD, USA

**Keywords:** substance use disorder, drug abuse, substance abuse, addiction, dependence, addiction treatment, substance abuse treatment, drug abuse treatment

## Introduction

Medically harmful substance use is common in the United States (U.S.), with an estimated 24.6 million Americans aged 12 years and older having used illicit drugs or engaged in non-medical drug use in the prior month, representing 9.4% of the population aged 12 years or older (1). Marijuana is the most commonly used illicit drug. In 2013, there were 19.8 million current users aged 12 years or older, 7.5% of the population in this age group (1). However, in 2013, only 2.5 million individuals in the U.S. received treatment at a specialty facility for an illicit drug or alcohol use problem in the past year, similar to numbers from 2002 through 2012 (1). Therefore, the vast majority of persons with substance use disorders (SUD) are not seen by specialty programs, and efforts to integrate SUD care with primary care in general medical settings have largely fallen short. Drug use disorders produce a wide variety of medical problems and are important contributors to years of life lost due to disability (2). A common barrier to treating individuals with cannabis use disorders (CUD) and other SUD in medical settings or successfully linking them to indicated follow-up care is inability of clinicians to engage patients in a collaborative dialog concerning the medically harmful consequences of unhealthy substance use and evidence-based treatment options aligned with patient values. Clinicians often do not use a shared-decision-making approach to discuss with patients’ different options for care personalized to risk categories and their preferences (3). Indeed, patients are often offered a single or narrow set of options for follow-up care, compromising their motivation to change their medically harmful substance use as well as engagement and initiation of treatment.

Here, I illuminate a need for systematic research in medical settings to evaluate a patient-centered model for treating CUD and other SUD, which incorporates principles of shared-decision-making engaging both patients and clinicians, grounded in personalized-medicine tailored to substance use risk categories and individualized patient values/preferences. Shared-decision-making and patient-centered care (4), considering individual preferences for treatment options, are critical for patient engagement in substance use disorder care. They necessitate a collaborative dialog between patients and providers in which they discuss benefits and risks of different evidence-based treatments, as well as the importance of patients in making decisions about their care. The provider helps patients understand their medical condition in a manner in which they feel empowered to make decisions about options for evidence-based care aligned with their values, in the spirit of personalized medicine (5). Medical setting providers need to evaluate: (1) substance use disorder severity, (2) presence of co-occurring psychiatric and other medical conditions, (3) readiness of patients to change their medically harmful substance use, (4) reasons for this readiness or hesitancy to do so, and (5) how they may help patients in instituting an action plan consistent with their preferences (5).

Rigorous research is needed in general medical settings testing effectiveness of primary and secondary prevention interventions incorporating principles of shared-decision-making and patient-centered care for individuals with multiple SUD (marijuana, alcohol, tobacco, or other commonly abused substances). This research is particularly needed in adolescents and young adults (age range 12–25 years old), where abuse of marijuana and prescription drugs has escalated in recent years (1). Important features of primary and secondary prevention interventions to be tested are: (1) be simple so they could be routinely delivered by clinicians at medical settings without cumbersome training and fidelity requirements, (2) utilize health information technology together with clinical decision support (CDS) tools to extend role of clinicians and simplify care delivery, (3) be either integrated with or utilize health information collected from electronic health records (EHRs), (4) use validated and brief electronic screening and brief assessment tool(s) for identifying risk categories of commonly abused substances, in a comprehensive way, (5) be sustainable and easily disseminated at conclusion of trials to applicable medical settings and stakeholders, (6) explain means of scalability and sustainability of effective secondary preventions, (7) include cost-effectiveness or other relevant cost–benefit analyses.

## The ASPIRE Framework for Patient-Centered Treatment Research

Recognizing a spectrum of neurobiological components for cannabis and other SUD, below I propose the ASPIRE model for patient-centered treatment, which uses as its foundational principle shared-decision-making to tailor *personalized* medical care to particular risk categories and problems that individual patients report as most distressing to their daily lives. “A” refers to anhedonia/reward-deficit and “S” to a stressful state, concerning a sensitized brain anhedonia and stress system following repeated heavy drug use and during drug withdrawal. According to prominent addictive-disorder researcher George Koob and over 30 years of cumulative neuroscience research by his team and others, this sensitized brain system that produces a negative emotional state in which motivation to remove it drives continued drug-taking behavior (6). This is analogous to Koob’s view of a “reward-deficit/stress surfeit” component of SUD in which “the negative emotional state is … mediated not only by deficits in the brain systems that mediate positive reinforcement but also by recruitment of brain stress/dysphoria systems that mediate negative reinforcement” (6). “P” refers to pathological lack of self-control to cut down drug use despite undesirable consequences. The compulsive drug use is hypothesized to involve impaired cortical regulation of impulses to reduce drug-taking despite negative implications (6). Neuroscience research over the past 25 years suggests that this lack of self-control is primed by a hypersensitive memory-trace of the reinforcing drug experience, which subconsciously drives compulsive drug seeking when coming in contact with drug-associated contexts (6). “I” and “R” refer to insomnia and restlessness, which are common cannabis withdrawal symptoms following repeated heavy drug use (7–9). Indeed, insomnia and restlessness are often rated by individuals with cannabis dependence as distressing and persistent withdrawal symptoms, which increase likelihood of relapse and hinder quit attempts (7–9). “E” refers to excessive and compulsive preoccupation with seeking drug reinforcers compared with natural reinforcers after transition from volitional to compulsive drug use, particularly following drug craving when an individual with a substance use disorder comes in contact with a drug-associated environment (6). In summary, systematic research is needed to test effectiveness of shared-decision-making and patient-centered care according to patients’ risk categories and reporting of role of components in the ASPIRE framework to their overall functioning and motivation for continued drug use. I include discussion of CUD as an illustrative example. However, the ASPIRE model is generalizable to patient-centered care for other SUD, too. For instance, insomnia and restlessness are common withdrawal symptoms for other SUDs such as nicotine, alcohol, opioid, and cocaine use disorders, which patients often find particularly distressing to their overall functioning and daily lives (10–13). In addition, the A, S, P, and E components are fundamental to the neurobiology of other SUDs (6, 14–16). Thus, across SUDs, it would be helpful for clinicians to ascertain these components’ role in a patient’s overall health and well-being, tailoring treatments according to the shared-decision-making model. Clinicians could then collaboratively and transparently engage their patients in instituting personalized action plans on how to best address them, weighing different evidence-based treatment options according to their clients’ preferences.

Furthermore, for patients at the high-severity end of the SUD spectrum, clinical research is needed testing effectiveness of combined behavioral and pharmacological interventions targeting prominent components in the ASPIRE framework. For CUD, evidence-based behavioral interventions include motivational enhancement therapy, cognitive–behavioral therapy, community-reinforcement approach, contingency management, and behavioral therapies aimed for adolescents (17). Pharmacological agents showing promise in proof-of-concept clinical research by Margaret Haney’s and Barbara Mason’s teams (18–20), meriting randomized controlled trials testing their effectiveness in treatment seekers, include: (1) noradrenergic alpha-2 receptor agonists (such as lofexidine or guanfacine) together with delta-9 tetrahydrocannabinol (THC) replacement therapy (such as nabilone) to reduce cannabis use, sleep disturbances, restlessness, anhedonia, and other prominent cannabis withdrawal symptoms, and (2) gabapentin for decreasing cannabis use as well as persistent cannabis withdrawal symptoms, such as craving and mood and sleep disturbances. In addition, the National Drug Abuse Treatment Clinical Trials Network of the National Institute on Drug Abuse (NIDA CTN) is presently conducting a multi-site clinical trial evaluating the efficacy of *N*-acetylcysteine versus placebo, added to contingency management, for cannabis cessation in adults aged 18–50 years old. The basis for this double-blind randomized controlled trial is positive findings from a randomized controlled trial in cannabis-dependent youth, which found more than twice greater odds of cannabis abstinence during treatment with *N*-acetylcysteine compared with placebo (21).

Since ASPIRE framework components generalize to treatment for other drugs of abuse, randomized clinical trials are also needed evaluating efficacy of pharmacological agents targeting ASPIRE components frequently reported to cause psychological distress in treatment-seeking patients. For instance, a recent randomized clinical trial from Barbara Mason’s group suggests that gabapentin treatment may be efficacious in reducing sleep disturbances and alcohol use and craving in alcohol-dependent patients (22), and an additional clinical trial is needed to replicate this. Clinical research from various groups suggests that noradrenergic alpha-2 receptor agonists (clonidine, guanfacine, lofexidine) may be efficacious in reducing stress and stress-induced craving for cocaine and opioids (23–25), and randomized clinical trials are needed to test whether these agents could also enhance abstinence from drug use in treatment seekers. In this regard, the ASPIRE framework may be useful to query patients prior to randomization on whether they experience individual components that these pharmacologic interventions are postulated to impact (e.g., stress, insomnia, restlessness). If so, the likelihood of detecting an efficacy signal may be improved by employing a personalized-medicine approach. In other words, patient subgroups that may benefit most from these agents would be included in randomized trials, according to the U.S. Food and Drug Administration’s (FDA’s) guidance on enrichment strategies for clinical trials to support approval of human drugs and biological products (26).

## Conclusion

Recent U.S. healthcare reform legislation provides unprecedented opportunities for SUD treatment to be integrated into general medical settings (27). This expansion of health care use by patients with SUD, under the 2010 Patient Protection and Affordable Care Act (ACA) and 2008 Mental Health Parity and Addiction Equity Act (MHPAEA), calls for new transformative research to guide evidence-based practices for SUD care in medical settings (27). Key research gaps need to be addressed concerning how to most effectively implement patient-centered personalized care incorporating shared-decision-making to engage patients as advocates in their treatment. The ASPIRE model described herein calls for personalized-medicine research on how to most effectively tailor evidence-based interventions in medical settings to address problem categories, which patients report as distressing to their daily lives. Among these may be core components of addictive disorders described in this framework. Clinical research programs, such as the NIDA CTN, can serve an important role in this endeavor. Leveraging existing health information system infrastructures in practice-based research networks is needed to accelerate this line of research. This could enable cost-efficient enrollment of patients into trials, recruitment of providers to deliver interventions within research studies, and utilization of existing health information technologies, including EHRs, mobile-health/telemedicine technologies, and EHRs-linked patient registries, for efficient clinical data collection to conduct low-cost-efficient effectiveness research.

## Conflict of Interest Statement

The author declares that the research was conducted in the absence of any commercial or financial relationships that could be construed as a potential conflict of interest.
